# Cerebellar cognitive affective syndrome (Schmahmann syndrome) following isolated cerebellar infarction

**DOI:** 10.1590/1980-5764-DN-2026-0500

**Published:** 2026-08-24

**Authors:** Mochamad Iskandarsyah Agung Ramadhan, Diatri Nari Lastri, Yetty Ramli, Pukovisa Prawiroharjo

**Affiliations:** 1 Universitas Indonesia, Faculty of Medicine, Department of Neurology, Jakarta, Indonesia.; 2 Dr. Cipto Mangunkusumo Hospital, Jakarta, Indonesia.

**Keywords:** Cerebellar Cognitive Affective Syndrome, Brain Infarction, Cognitive Dysfunction, Síndrome Afetiva Cognitiva Cerebelar, Infarto Encefálico, Disfunção Cognitiva

## Abstract

The cerebellum plays an important role in higher-order cognition through cerebrocerebellar networks. Lesions affecting this structure may result in cerebellar cognitive affective syndrome (CCAS), which is frequently underrecognized by routine cognitive screening tools. We reported a 69-year-old right-handed man who presented with 18 months of forgetfulness, impaired concentration, and irritability following a documented left cerebellar infarction. Neurological examination revealed cerebellar signs without cortical deficits. Brain magnetic resonance imaging (MRI) demonstrated a left cerebellar infarct with hemorrhagic transformation. While the Mini-Mental State Examination (MMSE) was normal and the Montreal Cognitive Assessment (MoCA) showed mild impairment, detailed neuropsychological assessment using CCAS Scale (CCAS-S) confirmed definite CCAS. This case highlights the importance of cerebellum-specific cognitive assessment in patients with isolated cerebellar stroke, as conventional screening tools might underestimate multidomain cognitive and affective disturbances.

## RESUMO

O cerebelo desempenha um papel importante na cognição de ordem superior por meio das redes cerebrocerebelares. Lesões que afetam essa estrutura podem resultar na síndrome cognitiva afetiva cerebelar (CCAS), que frequentemente não é reconhecida pelas ferramentas de triagem cognitiva de rotina. Relatamos o caso de um homem destro de 69 anos que apresentava, há 18 meses, esquecimento, dificuldade de concentração e irritabilidade após um infarto cerebelar esquerdo documentado. O exame neurológico revelou sinais cerebelares sem déficits corticais. A ressonância magnética cerebral demonstrou um infarto cerebelar esquerdo com transformação hemorrágica. Embora o Miniexame do Estado Mental (MMSE) estivesse normal e a Avaliação Cognitiva de Montreal (MoCA) mostrasse comprometimento leve, uma avaliação neuropsicológica detalhada utilizando a Escala de CCAS (CCAS-S) confirmou a presença definitiva de CCAS. Este caso destaca a importância da avaliação cognitiva específica do cerebelo em pacientes com acidente vascular cerebral cerebelar isolado, uma vez que as ferramentas convencionais de triagem podem subestimar distúrbios cognitivos e afetivos em múltiplos domínios.

## INTRODUCTION

The cerebellum’s role in cognition has long been underappreciated compared with cortical and subcortical structures. Growing evidence supports its role in higher-order cognition via extensive cerebrocerebellar networks^1,2^. Isolated cerebellar lesions may therefore produce cognitive and affective disturbances termed cerebellar cognitive affective syndrome (CCAS), or Schmahmann syndrome. Vascular lesions, including isolated cerebellar infarction, are recognized causes of this condition^3^. The cognitive presentations of CCAS might differ from cortico-subcortical deficits, and are often overlooked by routine neurocognitive screening tools such as the Mini-Mental State Examination (MMSE) or the Montreal Cognitive Assessment (MoCA)^4^. This case report describes an isolated cerebellar infarction presenting with cognitive and subtle affective symptoms.

## CASE PRESENTATION

A 69-year-old right-handed Minahasan man presented to the memory clinic with forgetfulness and impaired concentration for approximately 18 months. Symptoms began shortly after hospitalization for acute persistent dizziness, during which magnetic resonance imaging (MRI) confirmed an ischemic stroke. He was hospitalized for four days. Following discharge, he experienced difficulty retaining newly learned information, frequently forgot names and misplaced objects, and repeatedly asked the same questions. He became easily distracted while reading and driving. His wife reported increased irritability and emotional lability, including shouting in frustration when unable to recall information and snapping at family members when being reminded that he had asked the same question repeatedly.

Five years earlier, he had suffered an ischemic stroke presenting with dysarthria, right facial paresis, and right-sided weakness, which resolved without residual deficits. He held a bachelor’s degree in engineering and was retired from his profession as a contractor. Vascular risk factors included atrial fibrillation and controlled hypertension. His mother had late-life undiagnosed cognitive decline. Current medications were clopidogrel, warfarin, atorvastatin, and antihypertensives.

**Figure 1:**
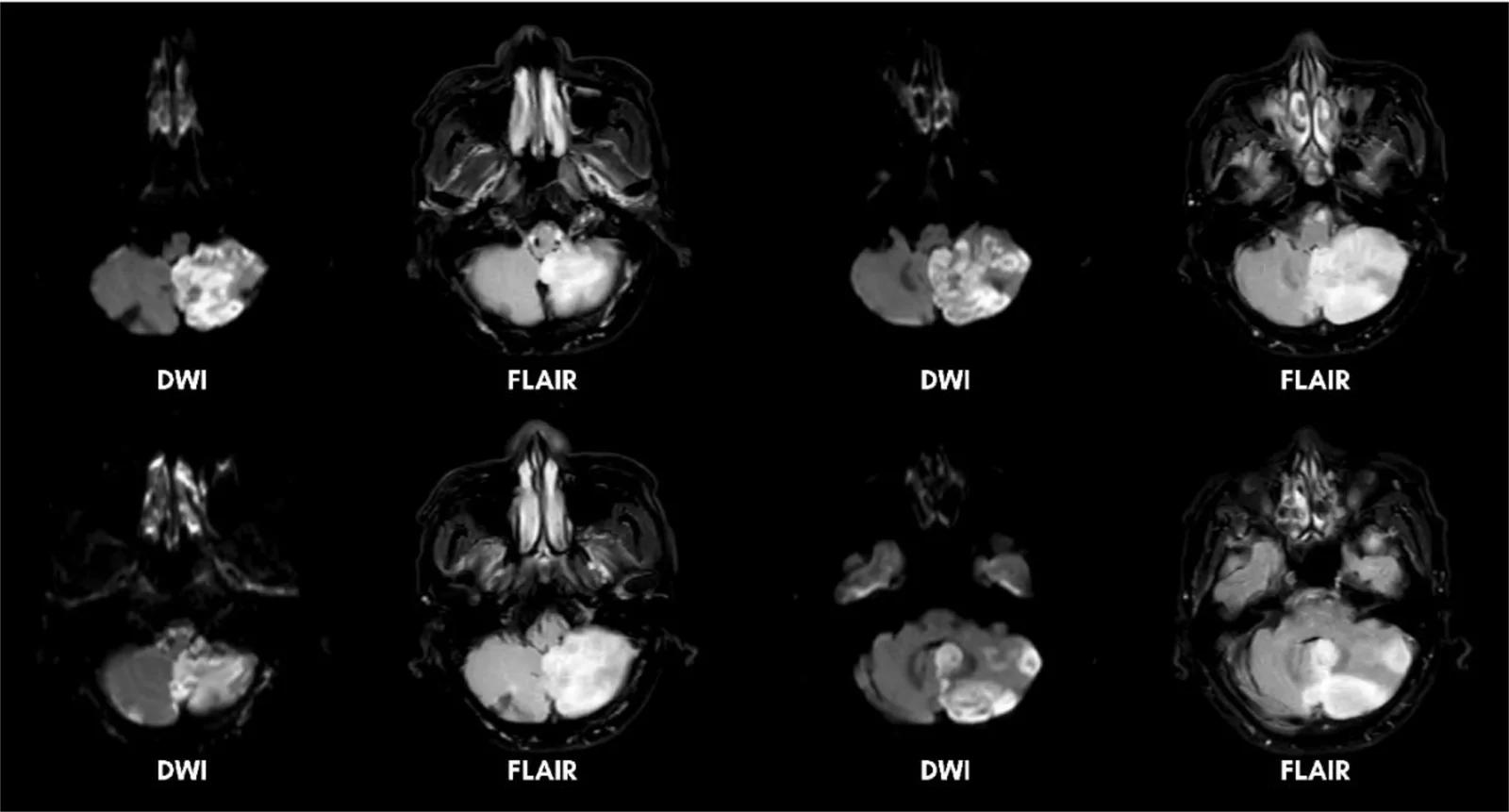
Non-contrast enhanced brain magnetic resonance imaging (MRI) showing a diffusion-restricted lesion on diffusion-weighted imaging (DWI) with hyperintensity and partial hypointensity on T2-weighted fluid-attenuated inversion recovery (T2W FLAIR) in the left cerebellum.

**Figure 2:**
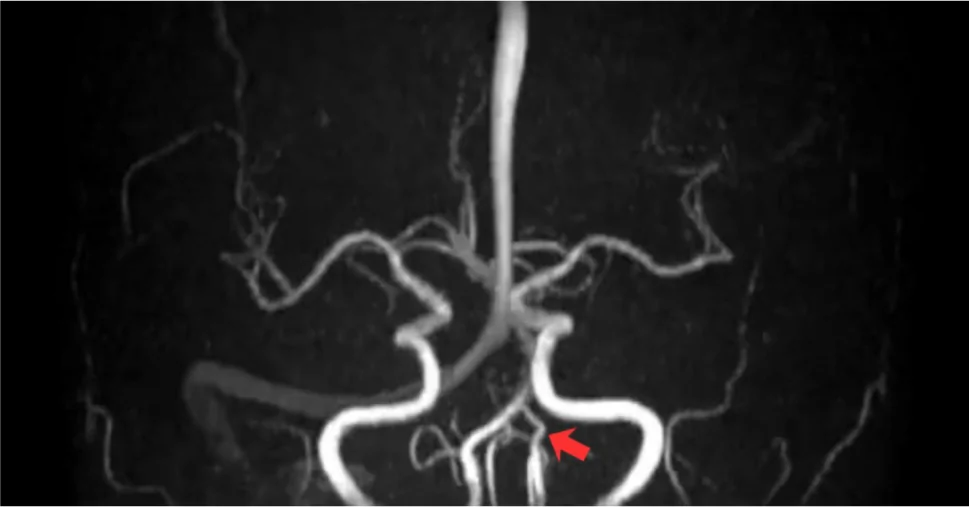
Magnetic resonance angiography (MRA) of the brain demonstrating diminished flow signal in the left posterior inferior cerebellar artery (PICA, red arrow).

Neurological examination revealed left-beating horizontal nystagmus and dysdiadochokinesia. He fell during sharpened Romberg and tandem gait testing, and could not complete the Fukuda stepping test due to imbalance. No pyramidal or cortical signs were observed. Brain MRI during hospitalization demonstrated a diffusion-restricted lesion in the left cerebellum, hyperintense on diffusion-weighted imaging (DWI) and fluid-attenuated inversion recovery (FLAIR) (Figure 1), consistent with left cerebellar infarction with hemorrhagic transformation. Additional chronic infarcts were noted in the right cerebellum and pons. Periventricular white matter hyperintensities (WMH), consistent with chronic small vessel disease (CSVD) Fazekas grade II, were also present. Magnetic resonance angiography (MRA) showed distal left vertebral artery hypoplasia (Figure 2).

His further cognitive assessment showed a normal score of MMSE and decreased score of the Indonesian version of MoCA (MoCA-Ina). The Consortium to Establish a Registry for Alzheimer’s Disease (CERAD) battery showed impaired verbal fluency, reduced immediate memory, absent delayed recall, and poor recognition. The Trail Making Test Part A and B were within normal limits (Table 1). To further characterize cerebellar-related deficits, the CCAS/Schmahmann Scale (version 1A) was translated into Indonesian for clinical use. Slight cultural adjustments were introduced, such as using the letter “S” for the phonemic fluency task and replacing “Robert” with “Joko” in the recall item. The patient failed four components: phonemic fluency, category switching, digit span forward, and verbal recall, hence definite CCAS was confirmed. In the context of affective items, the patient scored on the item ‘Angry or aggressive, irritable, oppositional, difficulty with social cues and social boundaries.’

**Table 1:** Summary of Cognitive Assessment by Instrument.

Instrument	Findings	Interpretation
MMSE	27/30 (orientation −1, calculation −2); other domains intact	Within normal limits; mild calculation deficit
MoCA-Ina	20/30; impaired visuospatial/executive (4/5), attention (1/2), letter vigilance (0/1), calculation (2/3), repetition (1/2), delayed recall (0/5); preserved naming (3/3), abstraction (2/2), orientation (6/6), verbal fluency (1/1)	Impairment with executive and memory predominance
CERAD	Impaired verbal fluency (10 words/min), immediate recall (2/6/5), delayed recall (0/10), recognition (4/10 hits; 8 false positives); other language functions relatively preserved	Verbal memory and fluency impairment
TMT-A/B	TMT-A 33 seconds; TMT-B in 123 seconds with three errors	Within normal limits
CCAS-S (v1A)	Failed: phonemic fluency (6/19), category switching (4/15), digit span forward (4/8), verbal recall (10/15); Preserved: semantic fluency (16/26), digit span backward (4/6), cube drawing (15/15), similarities (7/8), go/no-go (1/2), affect (5/6; irritability noted)	Definite CCAS (≥3 failed items)

Abbreviations: MMSE, Mini-Mental State Examination; MoCA-Ina, Montreal Cognitive Assessment — Indonesian version; CERAD, Consortium to Establish a Registry for Alzheimer’s Disease; TMT, Trail Making Test; CCAS-S, Cerebellar Cognitive Affective Syndrome Scale (version 1A).

**Figure 3:**
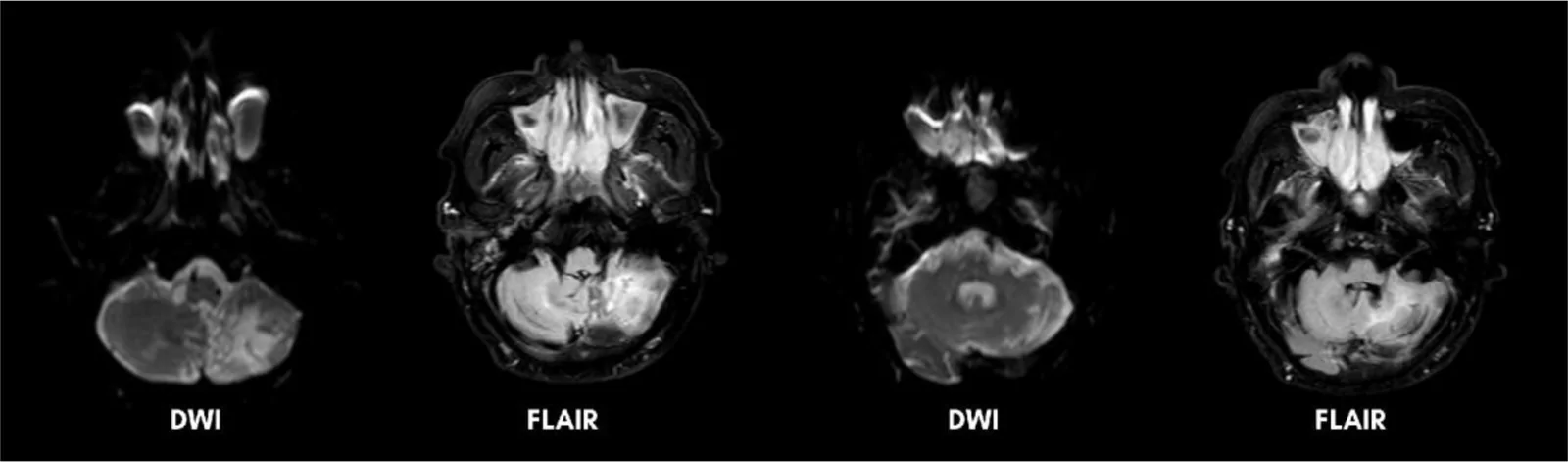
Follow-up non-contrast enhanced brain magnetic resonance imaging (MRI) at 1.5 years demonstrating no diffusion restriction on diffusion-weighted imaging (DWI), with a predominantly hypointense lesion and a hyperintense rim on T2-weighted fluid-attenuated inversion recovery (T2W FLAIR), consistent with a chronic cerebellar infarction, predominantly involving the left cerebellar hemisphere.

Based on the results of the aforementioned batteries, impairments were observed across multiple domains, including attention; language (spontaneous speech and repetition); memory (immediate recall, delayed verbal and visual memory, and recognition); and executive function (calculation and processing speed). Follow-up MRI performed approximately 1.5 years later for recurrent vertigo demonstrated no new infarction, with stable appearance of the prior cerebellar lesion (Figure 3). However, WMH had progressed from Fazekas grade II to grade III. Despite these radiological changes, no significant subjective cognitive decline was reported by the patient or caregiver over time.

## DISCUSSION

In 1997, Schmahmann and Sherman described 20 patients with focal cerebellar lesions who exhibited executive, visuospatial, linguistic, and affective disturbances, leading to the term CCAS, also known as Schmahmann Syndrome^2,4,5^. Cognitive impairment following cerebellar stroke exists along a spectrum, ranging from subtle executive dysfunction to the full CCAS phenotype. Isolated cerebellar infarctions account for approximately 1.65% of stroke cases, yet up to 84% of affected patients exhibit definite CCAS^3,4^. Smaller cohorts have demonstrated similarly high rates of cognitive and affective deficits in cerebellar infarcts, reaching 86 and 64%, respectively^6^. CCAS occurs more frequently in males and older adults, with lesions typically unilateral, slightly right-lateralized, and involving posterolateral regions linked to higher-order cognitive processing^3,7^.

Functionally, the cerebellum is divided into vestibular, motor, and cognitive ataxiology^2^. The cognitive cerebellum primarily involves the posterolateral hemispheres, particularly lobules VI and VII (Crus I–II and VIIB), in conjunction with the lateral dentate nucleus, while the posterior vermis contributes to affective and limbic regulation. Cerebellar cognitive function is mediated by cerebello-cortical loops, consisting of afferent cortico-ponto-cerebellar and efferent cerebello-thalamo-cortical pathways^2,8^. Infarction within the posterior inferior cerebellar artery (PICA) territory frequently involves these posterolateral regions, thereby disrupting cerebello-cortical networks and resulting in cognitive dysfunction^3,9^.

Two principal theories explain the pathomechanism of cognitive impairment in CCAS: the universal cerebellar transform (UCT), proposing uniform cerebellar microstructure applied across functional domains, and the dysmetria of thought (DoT) hypothesis, which conceptualizes cognitive impairment as analogous to motor incoordination^2,9,10^. Cerebellar functional lateralization parallels cerebral hemispheric asymmetry. Right-sided cerebellar lesions are more frequently associated with language impairment, whereas left-sided lesions tend to produce visuospatial deficits. Executive dysfunction, however, commonly involves bilateral cerebellar networks^9,11^.

Clinically, CCAS represents a multidomain cognitive disorder characterized by executive dysfunction, impaired attention shifting, reduced working memory, and slowed processing speed. Language disturbances, particularly reduced phonemic fluency, along with dysprosodia and agrammatism, are prominent, while visuospatial deficits affect higher-order construction and planning^2,4,9,10,12^. Although partial recovery may occur, deficits in attention, working memory, and executive language frequently persist, with additional impairments in category switching, digit span, and inhibitory control evident in the chronic phase^3,6,13^.

Affective symptoms are less well characterized but might include mood disturbance, irritability, and personality change, reflecting disruption of cerebellar-limbic circuits^2^. Notably, this patient exhibited irritability as the sole affective feature. Prior studies suggest affective symptoms were less frequent in acute cerebellar stroke, but might become more evident in chronic stages^3,13^. Lesion-based studies have linked left, medial, and right posterior cerebellar lesions with depressive symptoms, with prevalence influenced by age and vascular risk factors^14^. Nonetheless, this domain remains underexplored.

Although episodic memory impairment is atypical in CCAS, emerging evidence suggests cerebellar involvement. The observed deficits in immediate and delayed recall and recognition likely reflect disruption of cerebellar-hippocampal-prefrontal networks rather than primary hippocampal pathology^15^. Neuroimaging studies showed that episodic memory retrieval engages posterior cerebellar regions alongside hippocampal, prefrontal, and cingulate cortices, with regional cerebellar gray matter correlating with memory performance^16,17^. Neuromodulation study further demonstrated that anodal transcranial direct current stimulation of the right cerebellum enhanced episodic memory and hippocampal connectivity, supporting a causal role^16^.

Alternative causes, including hippocampal ischemia and neurodegenerative processes, were considered but were not supported clinically or radiologically. Furthermore, although CSVD was present and progressive, its contribution is likely limited, as it typically causes gradual decline in processing speed and executive function^18^. In contrast, our patient’s symptoms developed in temporal association with the cerebellar infarction and remained subjectively stable despite worsening CSVD radiological burden. While repeat neuropsychological testing is needed, the pattern argues against progressive vascular cognitive impairment and, instead, supports cerebellar network dysfunction following infarction.

Confirmation of CCAS requires domain-specific assessment. In 2018, Hoche et al^4^. introduced the CCAS Scale (CCAS-S), a 10-item battery assessing executive, language, visuospatial, memory, and affective functions, with defined cut-offs per item and a maximum score of 120. Failure of one, two, or three or more items indicates possible, probable, or definite CCAS, respectively. Unlike MMSE and MoCA, the CCAS-S applies stricter cut-offs and emphasizes cerebellar-specific features, such as cue-dependent recall and three-dimensional cube construction from verbal instruction alone. It demonstrates greater regional specificity, particularly for lesions in lobule VI, VII, and Crus I–II^4,13^.

Currently, no validated Indonesian version of the CCAS-S is available. The original CCAS-S has alternative forms with variations in item instructions and content, while the cube drawing and affective components remain unchanged. In the phonemic fluency task, different versions require participants to generate words beginning with the letters F, C, L, or B. Nevertheless, we used the letter S as it is more frequent and linguistically appropriate in the Indonesian language^19^. Additionally, Western names in the recall component (e.g., “Robert”) were replaced with culturally familiar Indonesian names (e.g., “Joko”) to enhance contextual relevance.

This case underscores the critical role of the cerebellum in cognitive processing. Cerebellar infarction might result in persistent multidomain cognitive impairment despite minimal supratentorial involvement. Recognition of CCAS, supported by the CCAS-S alongside MMSE, MoCA, CERAD, and the Trail Making Test (TMT), is essential for appropriate counseling and targeted rehabilitation. Further validation studies are warranted to establish the reliability and diagnostic accuracy of the CCAS-S in the Indonesian population.

## Data Availability

The datasets generated and/or analyzed during the current study are available from the corresponding author upon reasonable request.
